# Comparing internal migration across the countries of Latin America: A multidimensional approach

**DOI:** 10.1371/journal.pone.0173895

**Published:** 2017-03-22

**Authors:** Aude Bernard, Francisco Rowe, Martin Bell, Philipp Ueffing, Elin Charles-Edwards

**Affiliations:** 1 University of Queensland, Brisbane, Australia; 2 University of Liverpool, Liverpool, United Kingdom; 3 United Nations Population Division, New York, New York, United States of America; INDEPTH Network, GHANA

## Abstract

While considerable progress has been made in understanding the way particular aspects of internal migration, such as its intensity, age profile and spatial impact, vary between countries around the world, little attention to date has been given to establishing how these dimensions of migration interact in different national settings. We use recently developed measures of internal migration that are scale-independent to compare the overall intensity, age composition, spatial impact, and distance profile of internal migration in 19 Latin American countries. Comparisons reveal substantial cross-national variation but cluster analysis suggests the different dimensions of migration evolve systematically to form a broad sequence characterised by low intensities, young ages at migration, unbalanced flows and high friction of distance at lower levels of development, trending to high intensities, an older age profile of migration, more closely balanced flows and lower friction of distance at later stages of development. However, the transition is not linear and local contingencies, such as international migration and political control, often distort the migration-development nexus, leading to unique migration patterns in individual national contexts.

## Introduction

Migration is arguably the most complex component of demographic change, in part because of its multidimensional nature. Bell et al. argued that four discrete aspects of migration could be recognised, comprising migration intensity, impact, distance and connectivity, each of which provides a unique perspective on population movement [1]. They also proposed a series of robust metrics by which each facet of migration could be measured. Subsequent studies have applied these measures to explore cross-national variations with regard to overall intensities [2], age patterns [3], population redistribution [4] and distance moved [5], and have highlighted the substantial variations that exist between countries. For example, internal migration intensity measured over five years ranges from a low of 5 per cent in India to a high of 55 per cent in New Zealand [2], while the impact of migration on population distribution appears to follow a logistic curve with the development process [4]. Although these studies have provided valuable new insights into international variations in particular facets of movement, no concerted attempt has yet been made to examine inter-relationships between the various dimensions of movement and integrate them into a single encompassing framework. Simultaneous analysis across a large sample of countries should reveal whether different facets of migration are systematically related. Such analysis should also provide greater insights into the nexus between migration and development, as first sketched by Zelinsky [6], and thereby enhance understanding, explanation and migration theory. It may also offer new insights into migration patterns and processes within individual countries. At the same time, it is apparent that migration-development interactions are context-specific and migratory systems may therefore best be studied within a regional framework [7].

Latin America provides an ideal test-bed for such an integrated approach because of its low levels of international migration and fertility, which have resulted in internal population movement being the main driver of demographic change. Latin America also has the advantage of forming a distinct and coherent cultural region [8] founded on a common linguistic, religious and historical heritage. In addition, Latin American countries feature high levels of urbanization, similar settlement systems structured around primate cities [9], and export-oriented economies [10]. Despite these commonalities, evidence points to marked differences between countries across the region [11], on particular dimensions such as the intensity and age profile of migration. Latin America provides an ideal setting for exploring these different dimensions of migration in an integrated manner because countries in the region collect similar data, which avoids at least some of the problems of comparability that impede comparisons in other parts of the world [12]. Moreover, migration data for countries in the region are disseminated through a common portal via the REDATAM platform made available by the UN Economic Commission for Latin America and the Caribbean [13] and through the Integrated Public Use Microdata Series (IPUMS) database maintained by the Minnesota Population Centre [14]. These have subsequently been assembled as part of the Internal Migration Around the GlobE project (IMAGE), which assembled a global repository of global migration data, the IMAGE Repository [15, 16]. Coupled with recent methodological enhancements which circumvent differences between countries in geography and spatial frameworks [1], and development of the IMAGE Studio, a suite of analytical software to compute the system-wide migration indicators [17], this dataset provides a unique opportunity to systematically compare migration along multiple dimensions in different national settings.

In this paper, we draw on the IMAGE project’s data and methodological advances to compare countries on four dimensions of migration—intensity, age, impact and distance—and examine how they interact to generate migration signatures in 19 countries that cover 96 per cent of the population of Latin America and the Caribbean. In Section 2, we review past work on internal migration in Latin America and highlight the extent of cross-national variations on individual dimensions of migration. In Section 3, we discuss difficulties for cross-national comparisons arising from differences in data types, observation intervals and spatial frameworks, and present system-wide measures free of the influence of spatial scale. Section 4 explores how migration varies across 19 Latin American countries along each dimension alongside global averages. In Section 5, we use cluster analysis to explore how the four dimensions combine in particular country settings, and seek explanation for differences between clusters by reference to a range of economic, demographic and social indicators. We conclude by discussing how the different facets of migration co-evolve as development proceeds.

## Prior work

The spatial patterns of internal migration in many of the countries of Latin America are well documented. Considerable work has been undertaken to understand the dominant pattern of migration flows from rural communities to a small sub-set of large cities in Latin America during the 1940s and 1970s [18–20]. Capital cities were the main national centres of attraction over this period. In Argentina [21], Colombia [22] and Peru [23], for instance, Buenos Aires, Bogota, Guatemala and Lima reported the largest net migration gains, recording net migration rates of 31, 23, 23 and 38 percent during the 1960 and 1970 census rounds. Coupled with advances in transport infrastructure, this concentration of migration flows has been linked to the adoption of import substitution industrialisation policies which fostered concentration of economic activity and rapid urbanization in large Latin American cities [24, 25]

Less work has focused on more recent trends but several in-depth analyses have been undertaken. Recent studies have examined the intensity, spatial redistribution and demographic composition of internal migration flows. This work has focused particularly on Argentina [26], Brazil [27], Colombia [28], Chile [29, 30], Cuba [31] and Mexico [32]. Less attention has been given to the less developed countries of Central America and the Caribbean, such as Nicaragua, Honduras, El Salvador, Belize and Haiti.

The accumulated evidence highlights a number of similarities in contemporary internal migration patterns across Latin America [11, 33, 34]. First, the significance of rural-to-urban migration as a source of population growth in urban areas has diminished in most countries, with Bolivia experiencing the most significant reduction in the share of rural-to-urban migration to urban population growth from 64 percent in 1980–90 to 29 per cent in 1990–2000, and Mexico displaying the smallest drop from 33 to 32 percent [11]. Second, urban-to-urban migration has emerged as the dominant migration flow, reflecting increasingly high levels of urbanization [35, 36]. According to data from the 2000 census round, migration between urban areas accounted for over 50 percent of all internal population flows in Panama (51 percent), Paraguay (58 percent) and Brazil (62 percent) [11]. Third, the role of large cities as a primary destination for migrants has diminished, with large positive migration balances in the 1960s and 1970s falling to modest gains in countries such as Bolivia and Paraguay and reversing to net losses in Brazil [37], Chile [29] and Mexico [38] during the 1990s and 2000s [11], linked to the adoption of export-oriented development policies during the late 1970s and 1980s. These policies have triggered a dispersal of economic activity and population growth towards ports, borders and export-producing areas, particularly focusing on agriculture and mining outside large cities [9].

Cross-national variations in the intensity and spatial redistribution of internal migration in Latin America have also been examined (e.g., [11, 39, 40]). These studies have revealed remarkable variations in migration intensities with Chile, Antigua and Paraguay emerging as the most mobile countries, and Cuba, Nicaragua and Guatemala recording the lowest movement between administrative regions [11, 33]. These variations in migration intensity have resonated with marked differences in the spatial redistribution of migration [40], with Bolivia, Ecuador and Paraguay scoring the highest indices of migration efficiency, and Argentina, Chile, El Salvador and Nicaragua registering the lowest population exchanges [41].

While these studies have provided valuable insights into Latin American migration, research to date has been fragmented and confined to particular facets of population movement, such as overall intensities [11] and specific spatial processes such as urbanization [39] and counter-urbanization [42]. Existing studies have also been hindered by variations in the spatial framework against which migration is measured, giving rise to the modifiable areal unit problem (MAUP) [43]. In an attempt to address this issue, Rodriguez [44] distinguished migration according two levels of geographic scale—major and minor administrative regions—but the number of zones still varies widely from one country to the next. If full comparability is to be achieved, a more comprehensive approach is needed. A series of methodological advances developed through the IMAGE project now provide the tools to address these issues of comparability using scale-independent measures of migration intensity [2], impact [4] and distance [5], implemented using the IMAGE Studio which computes multiple random aggregations of geographic zones [17].

## Migration measures and data requirements

### Dimensions of migration

We focus on four dimensions of migration which together provide complementary insights into the dynamics of internal migration. These are (1) migration intensity, which indicates the overall level or incidence of migration within a country, (2) age patterns, which gauge the age composition of migrants, (3) migration impact, which captures the extent of population redistribution through migration, and (4) migration distance which indicates the effect of distance in constraining population movement. Bell et al. identified connectivity–the way migration serves to link cities and regions—as another dimension of migration [1], but for the purposes of this paper we confine attention to the four aspects listed above. Each of these can be captured using system-wide indices that provide summary measures at the national level. Table 1 lists the suite of measures and sets out the data required for their computation [15, 16] The measures themselves are explained in detail in Section 4.

**Table 1:** Migration indicators and data requirement.

	Migration measure	Data requirement			
		Population at risk	Data format		
			Origin-destination matrix	National count data	
				Total	by age
Intensity	Crude Migration Intensity* (CMI)	●	●	●	
	Aggregate Crude migration Intensity (ACMI)	●	●	●	
Age	Intensity at Migration Peak (IMP)	●			●
	Age at Migration Peak (AMP)	●			●
Impact	Migration Effectiveness Index (MEI)		●		
	Aggregate Net Migration Rate* (ANMR)	●	●		
	Index of Net Migration Impact (INMI)	●	●		
Distance	Distance Decay Parameter (*b)*		●+ digital boundaries		

* The CMI and the ANMR are both dependent on scale. Although they are not reported in this paper, they are listed here because they are used to derive the ACMI and the INMI as explained in Section 4.

Migration data are commonly available in two broad forms, both of which are used in this paper. Origin-destination matrices contain region-to-region migration flows, while count data comprise a single figure which indicates the total number of movers between regions within a country, irrespective of origin or destination, and are readily disaggregated by age. Allied to these forms of migration data, two other types of information are needed to compute some of the indicators. These are the population at risk, which is used to compute migration rates and probabilities, and digital boundaries identifying the regions against which migration is recorded, which are used to compute indicators of distance, calibrate spatial interaction models and drive the spatial aggregation facility in the IMAGE Studio. It is important to stress that the indicators listed in Table 1 are system-wide measures that provide national indices of intensity, age, impact and distance: they do not contain information on spatial patterns. As noted earlier, rural-urban migration and the role of migration in urbanization in Latin America are already well documented, so spatial patterns are excluded from this study.

Table 1 shows the data needed to generate the various migration metrics. With respect to intensity measures, for example, the Crude Migration Intensity, which is obtained by dividing the number of migrants by the population at risk, can be computed with migration data of any format, because it simply requires the aggregate number of migrants. This may be directly available in the form of a national migration count, but it can also be derived by summation from an origin-destination matrix. Other intensity measures require migration data disaggregated by age, which are more commonly held only in the form of nationwide migration counts. The computation of impact and distance measures requires data on inter-regional flows, from origin-destination matrices.

### Migration data

Comparability between countries is complicated by the fact that migration can be measured in various ways. All countries in Latin America collect migration data at their census [12], but they differ with respect to the type of data collected and the spatial frameworks employed, hindering cross-national comparisons [1]. While most Latin American countries collect data on migration transitions, derived by comparing place of residence at two points in time, some collect data on duration of residence, usually in association with a question on last place of residence irrespective of when the last move took place [12]. By filtering migration data for a given duration of residence, it is possible to derive surrogate estimates of the number of people who have moved within a given interval and hence establish migration flows broadly comparable to conventional migration transitions. To maximise the number of case study countries, we draw on transition data for 16 countries and on last residence data for 3 countries, Cuba, El Salvador and Panama. Migration transitions can be measured over any time interval, although the most common are one and five years, with the latter being most common in Latin America [12]. The longer the transition interval, the greater the potential effect of repeat and return migration, so migration intensities and migration flows measured over different length intervals cannot be reliably compared [45]. Because the size and composition of the population at risk of moving vary over time and because the contextual factors driving migration constantly evolve, five-year migration data provide a more realistic picture of the underlying migration flows than one-year data, which are affected by short-term fluctuations. To maximise comparability, we therefore estimate duration of residence at current place of residence over a five-year interval.

Even where countries collect the same type of data over equivalent observation intervals, comparisons are made difficult by differences in the number and arrangement of spatial units into which countries are divided. In Brazil, for example, the census collects data on migration between 1,504 municipalities, whereas in Costa Rica census data are available for movement between 81 cantons. Variations in the spatial scale at which migration is measured affect most measures of migration. A case in point is the Crude Migration Intensity (CMI) which increases with the number of spatial units [46] and therefore cannot be used to make direct cross-national comparisons. This bias is the product of the modifiable areal unit problem (MAUP), which plagues all geographic studies [43]. The measures used in this paper have the singular advantage of being scale independent and can therefore be used to compare countries even though some are calculated using different numbers of spatial units. With respect to intensity, we use the Aggregate Crude Migration Intensity, which overcomes the problem of spatial comparability by estimating all permanent changes of address [47]. While migration intensities have been shown to increase with the level of spatial disaggregation, the age profile of migration appears to be largely scale independent. Rogers and Castro found that the shape of the age profile of local mobility in the USA closely matched that of longer distance migration [48], and Bell and Muhidin reported a similar finding by comparing the age profiles of migration between minor administrative units in 19 countries with those of migration between major administrative units in the same countries [49]. There is accumulating evidence, however, that the age profile of migration varies widely between countries and over time, particularly with respect to the age at which migration peaks [49, 50]. With regard to measuring impact, the Aggregate Net Migration Rate (ANMR) originally proposed by Bell et al. [1] is dependent on spatial scale and so cannot be used to make cross-national comparisons directly. To circumvent this problem, Rees et al. [4] proposed a new Index of Net Migration Impact (INMI), which permits robust comparison with respect to the overall redistributive impact of migration. With respect to distance, Stillwell et al. [5] have empirically demonstrated for a global sample of 29 countries that the frictional effect of distance is relatively stable across spatial scale and can therefore be used to make direct comparisons across countries. The following section details the methodology used to compute each of these measures.

## Cross-national variation: Evidence from each dimension

This section explores how migration activity varies within Latin America across four discrete dimensions—intensity, age, impact and distance—using the indicators listed in Table 1. The method used to compute each indicator is presented first and estimated values are then reported for each country alongside global averages, which are based on samples of countries from all world regions, to provide a point of reference against which to interpret the results.

### Internal migration intensity

The crude migration intensity (CMI) is the simplest and most common measure of the overall propensity to move within a country and is analogous to the crude birth and death rates. For transition and last move data, it is expressed at the total number of internal migrants over a particular interval (M) as a percentage of the national population at risk of moving (P).

CMI=100M/P (1)

As noted earlier, the number of migrants recorded depends on the number and shape of spatial units into which a country is divided. Thus, cross-national differences in CMIs are, in part, a function of differences in spatial frameworks. An approach to circumvent the problem of spatial comparability is to compare countries with respect to all permanent changes of address [51] to obtain the aggregate crude migration intensity (ACMI). Very few countries, however, collect such data and none do so in Latin America [2]. To generate estimates for countries that do not collect such data directly, Courgeau et al. [47] proposed fitting a regression line to a series of CMIs measured at various spatial scales. The underpinning logic is that the number of migrants increases systematically with the number of zones into which a territory is divided. Courgeau et al. [47] demonstrate that setting the observed CMI against the average number of households per zone (H/n) at each level of zonation delivers a linear equation of the form:

$$CMI_{n}=w+k\text{ln}\left[H/n\right]$$ (2)

where *CMI*_*n*_ is the Crude Migration Intensity at the level of n zones, H is the national count of households, k is an empirically derived scaling factor and w is a constant. Substituting *H*/*n* = 1 in the estimated equation corresponds to a hypothetical level at which there is an average of just one household per zone, and any move between zones therefore represents a change of address. Since ln(1) = 0, the corresponding ACMI is simply represented by the constant, w. Eq 2 can be readily estimated from most national migration statistics provided that migration data are available for at least two levels of geography. However, greater precision is achieved by using estimates computed for geographies at multiple spatial scales, as facilitated by the randomised geographies facility implemented in the IMAGE Studio [17, 52]. Bell et al. provide a comprehensive overview of the methodology [2].

**Fig 1:**
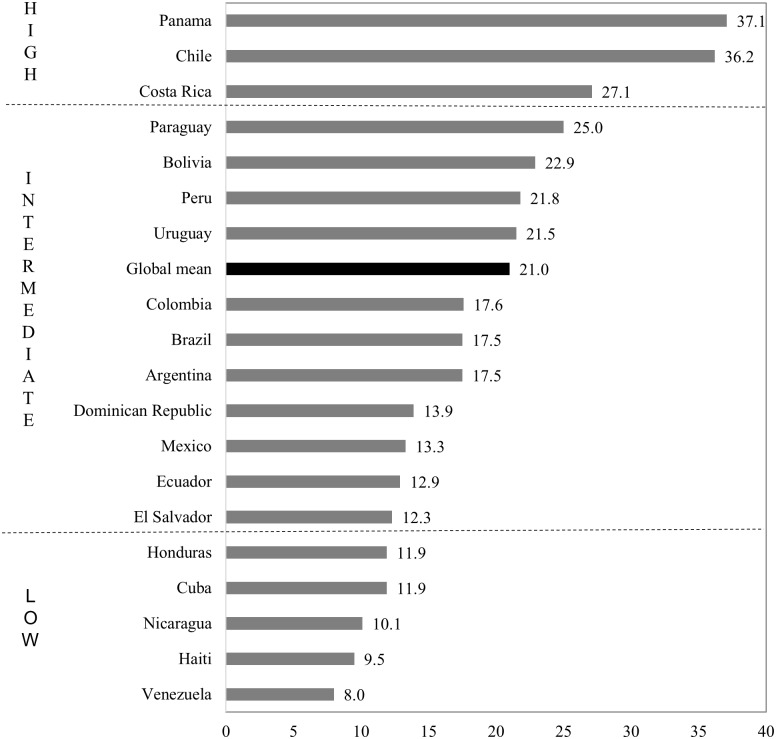
Aggregate crude migration intensity. Source: IMAGE Repository, global mean across sample of 61 countries encompassing all world regions from [2] Note: Intensities greater than or equal to 0.5 standard deviation above the global mean are classified as high, intensities 0.5 standard deviation above or below are classified as intermediate, and intensities lower than or equal to 0.5 standard deviation below the global mean are classified as low.

In this paper, we use the above techniques in association with the IMAGE Studio, to estimate the ACMI for 19 Latin American countries, which effectively delivers estimates of all changes of residential address over a five year interval that can be directly compared between countries. Fig 1 ranks the 19 countries from highest to lowest ACMI and reveals marked variations. Migration intensities vary from highs over 30 per cent in Chile and Panama to lows of less than 10 per cent in Haiti and Venezuela, with a sample mean of 18.5 per cent, which is broadly on par with the global mean. Migration intensities in Latin America are quite evenly distributed around the global mean. Eleven of the 19 countries display intensities within 0.5 standard deviations of the global mean, four feature high intensities while five register intensities at the lower end of the global scale. As observed by Bell et al. [2], the results reveal a marked spatial gradient across the continent trending from low intensities in Mexico, the less developed parts of Central America (Honduras and El Salvador) and the Caribbean (Haiti and Cuba) and Venezuela to moderate intensities in Argentina, Colombia and Brazil, culminating in high intensities in Chile and in the more developed countries of Central America (Panama and Costa Rica). The use of a scale-independent measure of intensity shows greater variation than had previously been observed in Latin America and a different rank order of countries. While previous research had positioned Panama and Costa Rica at intermediate levels of mobility, on a par with Ecuador, Uruguay and Venezuela [11], the current results show a significantly higher underlying propensity to move.

### Age patterns

**Fig 2:**
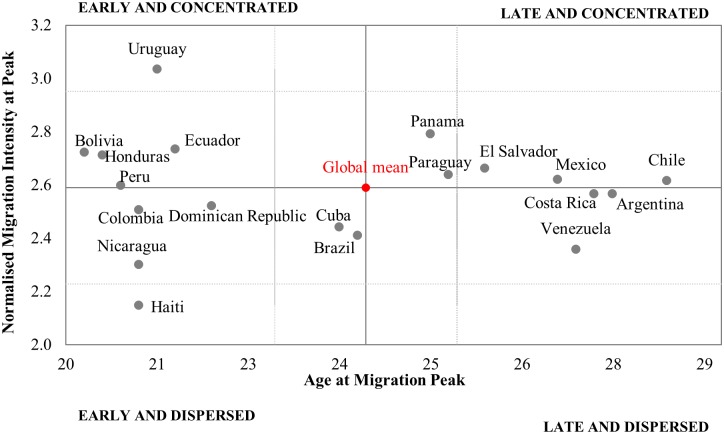
Age at migration peak against normalised migration intensity at peak. Source: IMAGE Repository. Note: Measured were derived from migration data disaggregated by single years of age, normalised to unity, and smoothed using Kernel regression [53]. Global mean was estimated for sample of 25 countries encompassing all world regions [3]. Gridlines are located 0.5 standard deviations from the global mean.

Despite broadly similar migration age profiles from one country to another [48] recent research shows that countries vary in the ages and level at which migration peaks [3]. These variations, combined with differences in population age structures, shape overall migration intensities. To systematically explore these variations in Latin America, Fig 2 reports two indicators that summarise migration age patterns: the age at which migration peaks, and the intensity of migration at the peak. In a comparative analysis of 25 countries, Bernard et al. demonstrated that about two thirds of the inter-country variance in migration age profiles is captured by these two measures [3]. They also have the advantage of having an intrinsic meaning. The age at which migration peaks captures how early in life migration occurs, while the intensity of migration at the peaks gauges the degree of concentration of migration activity at young adult ages. To uncover cross-national differences in the age structure of migration, the data has to be normalised to unity so that measures of age are independent from variations in overall intensities.

To provide a broader global context against which to interpret the results, the axes in Fig 2 intersect at a mean obtained from a sample of 25 countries as reported in [3] with gridlines located at 0.5 standard deviations. With respect to age at the migration peak, only four countries—Cuba, Brazil, Panama and Paraguay—lie within 0.5 standard deviations of the global mean. Other countries are strongly differentiated by age. Late migration peaks around 27 years are observed in Argentina, Costa Rica, Chile, Mexico and Venezuela, whereas the less developed parts of Central America and the Caribbean (Haiti, Honduras and Nicaragua) as well as Bolivia, Colombia and Peru feature early migration peaks at 20 or 21 years. However, the data reveal little variation in the extent to which migration is concentrated around the peak: except for Uruguay and Haiti, all countries fall within 0.5 standard deviations of the global mean. This profile of Latin American countries with respect to age at the migration peak distinguishes the region from other parts of the word, where migration age patterns tend to be more homogenous. European countries, for example, are characterised by late ages at the peak and dispersed profiles, whereas Asian countries predominantly feature early and concentrated age profiles [3].

### Impacts on population redistribution

Migration is inherently a spatial process which transforms the settlement system by redistributing the population between regions, either increasing or reducing the degree of population concentration in particular areas. While analysts have commonly focused on rural to urban migration, definitions of urban vary widely, and simple measures of urbanization fail to capture more subtle transformations of the settlement pattern. Bell et al. [1] proposed the Aggregate Net Migration Rate (ANMR) as a more comprehensive system-wide measure of the impact of net migration on the pattern of settlement within a country, defined as half the sum of the absolute net changes across all regions, divided by the population at risk *P*:

$$ANMR=100*0.5\sum_{I}\left|D_{i}-o_{i}\right|/P$$ (3)

where *D*_*i*_ and *O*_*i*_ are in- and out-flows from region *i*. The ANMR identifies the net shift of population between regions per 100 residents in the country and is a product of the Crude Migration Intensity defined in Eq (1) and the Migration Effectiveness Index (MEI) such that:

ANMR=CMI*MEI/100 (4)

where:

$$\;MEI=100*0.5\sum_{I}\left|D_{i}-O_{i}\right|/M$$ (5)

While the CMI measures the overall incidence of migration, the MEI captures its effectiveness as a mechanism for redistributing population by comparing net migration to migration turnover. It quantifies the spatial imbalance between migration flows and counter-flows, with low values indicating closely balanced flows and counter-flows while high values indicate greater asymmetry, with some regions gaining population at the expense of others. Because the ANMR is a product of the CMI, its value increases with the number of spatial units, and it therefore cannot be used to make cross-national comparisons directly. However, Rees et al. [4] show that both the CMI and the MEI are linear functions of the number of zones into which the territory is divided: while the CMI rises steadily as the zone count increases, the MEI is stable and largely scale independent above a threshold of 20 zones. Rees et al. [4] then demonstrate algebraically that the slope of the ANMR is a product of the slope of the CMI and the average level of the MEI, which provides a robust basis for making comparisons of migration impact between countries, irrespective of the number of regions used to measure migration. To facilitate comparisons, Rees et al. [4] recommend adopting the mean across a sample of countries as a point of reference and define the proposed new measure as the Index of Net Migration Impact (INMI), computed as:

$$INMI=\frac{CMI\;slope\;for\;a\;country}{Average\;CMI\;slope\;for\;all\;countries}*\frac{Mean\;MEIfor\;a\;country}{Average\;MEI\;for\;all\;countries}$$ (6)

As well as facilitating robust comparisons with respect to the overall redistributive effect of internal migration, the particular advantage of the INMI lies in distinguishing between the relative contributions of migration intensity and migration effectiveness.

**Fig 3:**
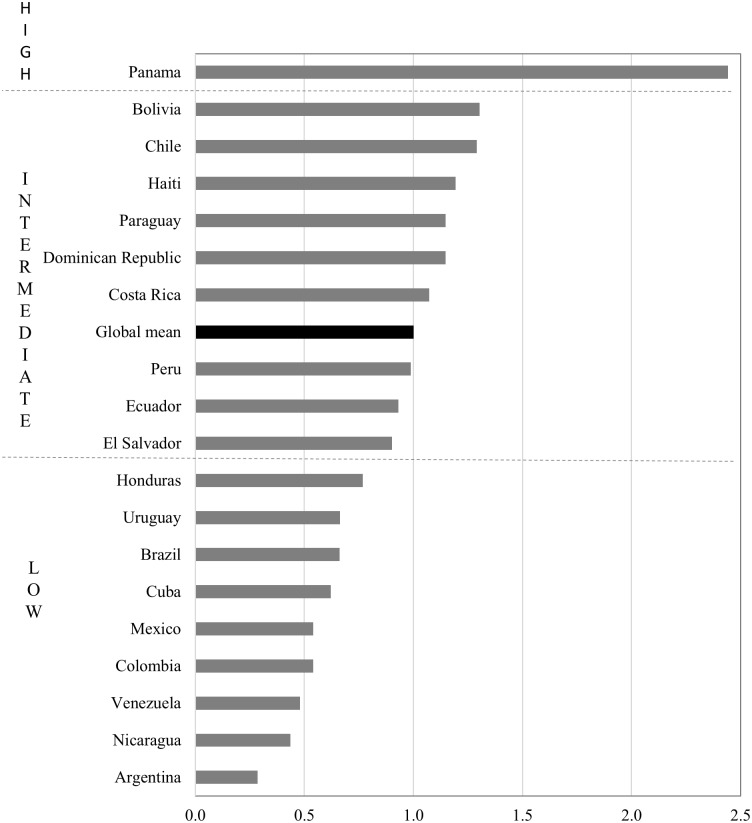
Index of Net Migration Impact. Source: IMAGE Repository, global mean across a sample of 71 countries from [4] Note: INMIs greater than or equal to 0.5 standard deviations above the global mean are classified as high, INMIs 0.5 standard deviations above or below are classified as intermediate, and INMIs lower than or equal to 0.5 standard deviations below the global mean are classified as low.

**Fig 4:**
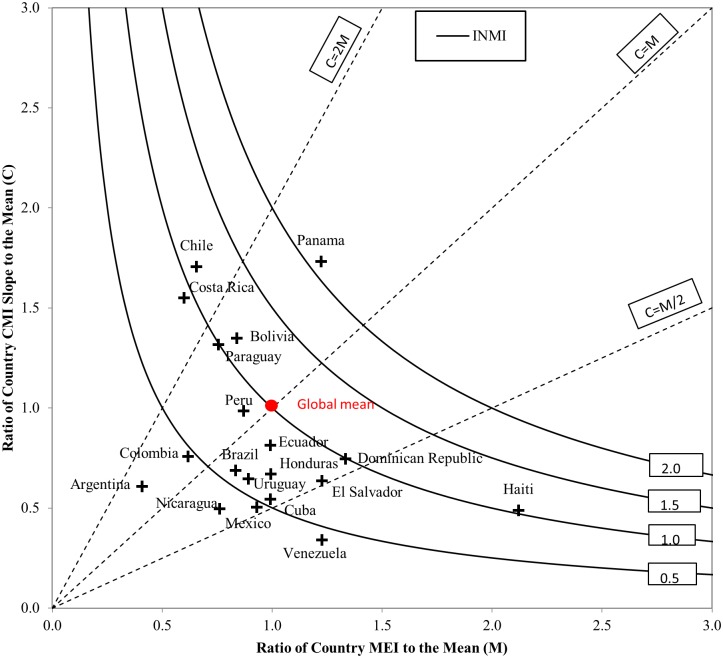
Migration effectiveness against migration intensity. Source: IMAGE Repository, global mean across a sample of 71 countries from [4].

For the present paper, we compute the INMI for 19 Latin American countries against the global sample of 71 countries encompassing all world regions as reported by Rees et al. [4] Fig 3 ranks countries with respect to the aggregate INMI, and Fig 4 plots the same values distinguishing its constituent components. Values above unity indicate that the effect of migration in redistributing population is greater than the global average, and vice versa. The results show that the impact of migration in redistributing population is relatively low in Latin America. Of the 19 countries, 12 display migration impacts below the global mean Migration impacts are lowest in Venezuela, Nicaragua and Argentina, where the INMI is less than half the global mean. Conversely Panama stands out with the highest level of population redistribution, twice the global average.

To distinguish the relative contributions of migration intensity and migration effectiveness to the INMI, Fig 4 displays the ratio of the CMI slope to the mean against the ratio of the Migration Effectiveness Index to the mean. The surface plot represents the INMI and the contour lines link points of equal impact. The results underline the complex interaction between intensity and effectiveness in driving population redistribution. In Panama, it is clear that the very high level of population redistribution is driven equally by above average levels of migration intensity and migration effectiveness. For both Chile and Haiti, the impact of migration in redistributing populations is lower, just a little above the global mean, but the underlying drivers are different. In Chile, population redistribution is due to high migration intensity, whereas in Haiti low migration intensity is compensated by very high migration effectiveness. Close inspection of Fig 4 suggests that four distinct regional groups can be identified: (1) countries where low effectiveness offsets high intensities, as in Chile, Costa Rica, Bolivia and Paraguay, (2) a cluster of countries slightly below the mean on both drivers, as in Peru, Ecuador, Colombia, Uruguay and Brazil, generating below average INMI, (3) a group of countries in Central America (El Salvador and Honduras) and the Caribbean (Dominican Republic, Cuba and Haiti) where low intensities strongly constrain the redistributive effect of migration, this is particularly pronounced in Haiti, and (4) countries with equally low migration intensity and migration effectiveness, as in Colombia, Argentina and Nicaragua. As noted by Rees et al. [4] there is a general tendency for high migration effectiveness to be offset by low migration intensity (Haiti) or, conversely, for low effectiveness to be offset by high intensity (Chile, Costa Rica, Bolivia and Paraguay). Only very few countries feature consistently high (Panama) or low values on both drivers (Argentina).

### Friction of distance

**Fig 5:**
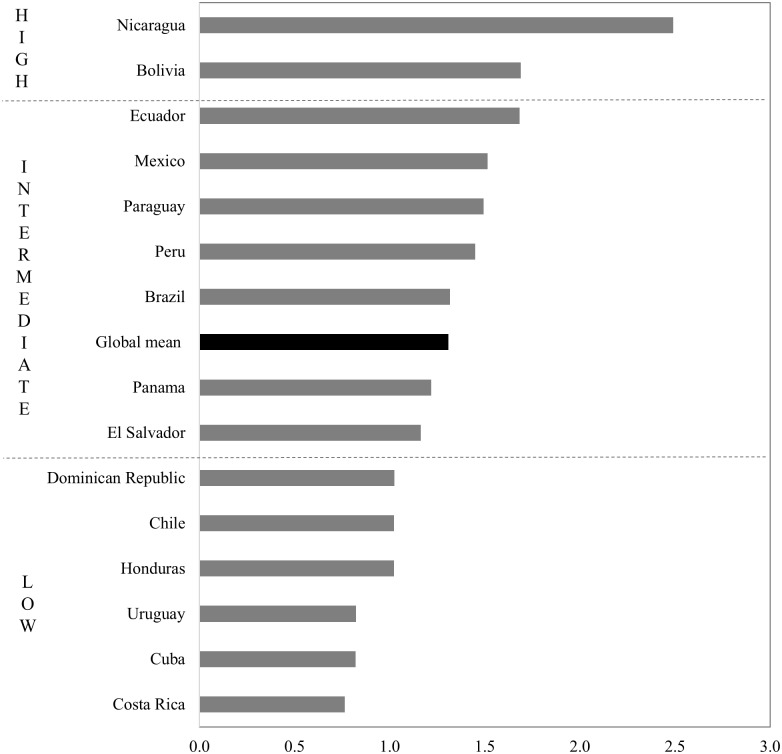
Distance decay parameter for regions with mean populations of 200,000. Source: IMAGE Repository, global mean across a sample of 29 countries from [5] Note: Distance decay parameters greater than or equal to 0.5 standard deviation above the global mean are classified as high, distance decay parameters 0.5 standard deviation above or below are classified as intermediate, and distance decay parameters lower than or equal to 0.5 standard deviation below the global mean are classified as low.

We now turn attention to the friction of distance, which measures the way in which distance acts as a deterrent to population movement. While distance decay effects have been measured for a number of individual countries, including Brazil [54], few attempts have been made at cross-national comparisons. Bell et al. [1] proposed a number of indicators, pre-eminent among which is the distance decay parameter, *β*, obtained by fitting a spatial interaction model. In the IMAGE Studio, it is estimated using an iterative search routine [17] where:

$$M_{ij}^{\prime }=A_{i}B_{j}O_{i}D_{j}d_{ij}^{-\beta }$$ (7)

where $M_{ij}^{\prime }$ is the modelled migration flow between region *i* and region *j*, *O*_*i*_ is the total of out-migrants from region *i* to all destinations, *D*_*j*_ is the total of in-migrants to region *i* from all origins, *A*_*i*_ and *B*_*j*_ are balancing factors to ensure that the predicted flows sum to *O*_*i*_ and *D*_*j*_, and *d*_*ij*_ is the distance between distance between region *i* and region *j*. Using a global sample of 29 countries, Stillwell et al. [5] have demonstrated empirically that the frictional effect of distance is relatively stable across spatial scales. Thus, the distance decay parameter estimated for regions of similar mean population sizes can be reliably compared between countries even though their spatial frameworks differ. Spatial interaction models need to be fitted at a spatial scale which is sufficiently fine to generate reliable estimates, so we restrict analysis to the 15 countries that collect migration data between 75 administrative units or more. Fig 5 reports the distance decay parameters computed when the model is run for regions with mean population sizes of 200,000. The results reveal substantial variation between countries. Nicaragua, Bolivia and Ecuador display high levels of distance friction, with distance decay parameters more than 0.5 standard deviations above the global mean, while Costa Rica, Cuba and Uruguay report low friction of distance, more than 0.5 standard deviations below the global mean. Brazil and Panama display parameters on par with the global mean.

### Integrating the dimensions of migration

Each facet of population movement provides a different picture of Latin America: the order of countries differs according to the dimension being examined. Overall intensities, for example, position Panama and Chile as the most mobile nations, with intensities double that of Argentina, and more than triple those of Haiti and Venezuela. Despite very different intensities, Chile and Argentina share late age patterns, which stand in contrast to the early age profiles of Haiti, Nicaragua, Honduras and Bolivia. A different picture emerges when considering the impact of migration. Opposing patterns distinguish Panama, which features high migration impact driven by high intensity and effectiveness, from Argentina and Colombia, characterised by low intensity and effectiveness. There are also substantial variations in the frictional effect of distance, with low friction in some parts of the Caribbean and Central America (Cuba, Dominican Republic, Costa Rica and Honduras) contrasted with moderately high friction in Brazil and Mexico. Correlation analysis in Table 2 confirms that the four dimensions of migration are distinct, with the exception of impact, which by construction is a product of effectiveness and intensity and therefore returns a strong association with the latter (r = 0.69). While intensity at the peak is also associated with overall intensity (r = 0.48), age at the peak, effectiveness and distance are clearly independent from the other dimensions of migration. Despite these modest correlations, the results suggest that certain aspects of migration evolve in parallel as development proceeds. Countries at higher levels of development are characterised by higher migration intensities, older ages at the migration peak, balanced flows and lower friction of distance, while countries at lower levels of development exhibit lower intensities, younger ages at the peak, unbalanced flows and higher distance friction. However, there are also marked anomalies, such as in the case of Argentina which displays a low level of migration intensity relative to its development. This suggests there is no simple linear association between the various facets of migration and national development.

**Table 2:** Pearson’s correlation coefficients between migration statistics.

	**Intensity (ACMI)**	**Age at Peak**	**Intensity at Peak**	**Effectiveness (Mean MEI)**	**Impact (INMI)**	**Distance (β parameter)**
**Intensity (ACMI)**	1					
**Age at Peak**	0.32	1				
**Intensity at Peak**	0.48*	-0.03	1			
**Effectiveness (Mean MEI)**	-0.36	-0.27	-0.35	1		
**Impact (INMI)**	0.69*	0.01	0.28	0.32	1	
**Distance (β parameter)**	-0.23	-0.37	0.04	-0.14	-0.20	1

* correlation significant at the 5% level

To untangle these connections, we bring together the different dimensions of migration by running a k-mean cluster analysis of the 19 countries, based on overall migration intensity, migration effectiveness, and age at the migration peak, all normalised to unit variance. To determine the appropriate number of clusters, we first performed a Ward hierarchical cluster algorithm and used the resulting data as input in a k-means cluster analysis. Migration impact was excluded because it is a product of intensity and effectiveness. Migration intensity at the peak was also excluded because of the limited variation in our sample while the beta parameter was available only for a subset of 15 countries. However, mean values for each excluded indicator are reported by cluster in the table of results (Table 3). The analysis delivered a four cluster solution.

**Table 3:** Migration statistic mean values by migration cluster.

	Cluster 1	Cluster 2	Cluster 3	Cluster 4
Countries	Chile, Costa Rica, Panama	Bolivia, Dominican Republic, Ecuador, Haiti, Honduras, Nicaragua, Peru	Argentina, Brazil, Colombia, Paraguay, Uruguay	Cuba, El Salvador, Mexico, Venezuela
**Intensity (ACMI)**	33.47	14.71	19.82	11.38
**Age at Peak**	26.83	21.00	23.80	25.81
**Effectiveness (mean MEI)**	21.53	29.45	18.27	28.52
**Impact (INMI)**	1.60	0.97	0.66	0.64
Distance (β parameter)*	1.00	1.56	1.21	1.17

Note: While the K-mean cluster analysis was ran on variables normalised to unit variance, migration measures reported in here are not standardised for ease of interpretation. Because this component of the analysis does not focus on placing Latin America in a global context, effectiveness is not reported here a ratio against the global mean, contrary to Fig 4.

* The β parameter is not available for Haiti (Cluster 2), Argentina and Colombia (Cluster 3), and Venezuela (Cluster 4)

A first group of countries—Chile, Costa Rica and Panama—exhibit high intensities and an old age profile, with a migration peak in the late twenties. These countries are characterised by low friction of distance and varied migration effectiveness, high in Panama but low in Chile and Costa Rica. The second cluster includes countries in Central America (Nicaragua and Honduras), the Caribbean (Dominican Republic and Haiti), and the Andes (Bolivia, Ecuador and Peru). It features a migration peak in the early twenties and low (Ecuador Dominican Republic, Haiti, Honduras and Nicaragua) to intermediate intensities (Peru and Bolivia), which strongly constrain the redistributive effect of migration despite high migration effectiveness. These countries also display high friction of distance. A third cluster encompasses five MERCOSUR countries, Argentina, Brazil, Uruguay, Paraguay and Colombia. This group features intermediate intensities and low effectiveness, resulting in very limited migration impact on the settlement system. These countries also exhibit intermediate migration peaks and moderate to high friction of distance. Cuba, El Salvador, Mexico and Venezuela form the fourth cluster because of a unique combination of low intensities and late age profiles. Migration in these countries also exerted very limited impact in terms of population redistribution, because of the combination of low intensities and moderate effectiveness.

Explanation for these differences in the migration experience of Latin American countries must be sought from a range of perspectives. Zelinsky’s original hypothesis of the mobility transition sought to relate changes in spatial mobility to stages of the demographic transition—the historical shift from high fertility and mortality which characterized traditional societies, to the modern era in which both processes return to balance at a lower level of intensity [6]. Recognizing that mobility is in reality a more complex, multifaceted process, we give consideration to a broader range of forces that can be grouped under four discrete headings: geographic, demographic, economic and social, all of which shape the way particular aspects of migration are transformed during the process of national development [2, 4, 5]. Recognizing the diversity of drivers, we seek to tease out the relationship between contextual factors and migration processes using a series of readily available national indicators that characterize the broader socio-economic context. To that end, we employ a two-stage analysis. First, we identify how contextual factors vary between clusters. We construct four binary variables, one for each cluster, and assign values to each of the 19 countries with 1 indicating cluster membership and 0 otherwise. We then correlate these variables with each national indicator. Secondly, in order to provide a more finely grained analysis of how the socio-economic context shapes each dimension of migration, we then run a second set of correlation analyses between each national indicator and each migration measure. We discuss the outcomes below and report the detailed results of these analyses in Tables 4 and 5.

**Table 4:** Pearson’s correlation coefficients between cluster membership and national indicators.

	Cluster 1	Cluster 2	Cluster 3	Cluster 4
	Chile, Costa Rica, Panama	Bolivia, Dominican Republic, Ecuador, Haiti, Honduras, Nicaragua, Peru	Argentina, Brazil, Colombia, Paraguay, Uruguay	Cuba, El Salvador, Mexico, Venezuela
**Geographic**				
Area	-0.20	-0.31*	0.53***	-0.04
Population density (2000)	-0.12	0.23	-0.36***	0.23
Urbanization (2000)	0.06	-0.60**	0.39*	0.23
**Economic**				
GDP per capita (2005 PPP$)	0.36**	-0.73***	0.21	0.33
Gini index (2000,2005)	-0.06	0.15	0.03	-0.17
Foreign direct investment % of GDP (2000)	0.08	0.14	0.05	-0.32**
Employment in agriculture (%) (2005)	-0.21*	0.64***	-0.29	-0.26*
Labour force participation (2000)	-0.42***	0.32	0.22	-0.24
**Social**				
HDI (2000 and 2005)	0.40***	-0.63***	0.26	0.11
Mobile telephone subscribers 2000	0.41***	-0.72***	0.50***	0.00
Literacy (2000)	0.25**	-0.55***	0.26**	0.15
Mean years of schooling (2005)	0.45***	-0.37*	-0.07	0.07
Homicide Rate (2005)	-0.27***	-0.03	-0.06	0.35
Political Rights (2010)	0.44***	-0.06	0.22	-0.56***
Civil Liberties (2010)	0.50***	-0.18	0.24	-0.49**
**Demographic**				
Population growth rate (2000–2005)	0.26	0.32*	-0.18	-0.41*
Life expectancy at birth (2000–2005)	0.48***	-0.57***	0.06	0.18
Total Fertility Rate (200–2005)	-0.28*	0.67***	-0.17	-0.35**
Median age (2000)	0.26	-0.58***	0.28	0.15
Mean age at first union (2000)	0.08	-0.46**	0.37	0.09
International net migration rate (2000–2005)	0.57***	-0.27	0.12	-0.32
Remittances as percent of % GDP (2000)	-0.37***	0.50**	-0.31**	0.07

Significance levels were derived from bootstrapped confidence intervals using 1000 replicates.

* Correlation significant at the 10% level,

** 5% level and

*** 1% level

**Table 5:** Pearson’s correlation coefficients between each migration measure and national indicators.

	Intensity (ACMI)	Age at Peak	Effectiveness (mean MEI)	Impact (INMI)	Distance (*β*parameter)
**Geographic**					
Area	0.01	0.23	-0.40**	-0.16	0.30
Population density (2000)	-0.39***	-0.10	0.78***	-0.26**	-0.28
Urbanization (2000)	0.21	0.45**	-0.50**	-0.08	-0.27
**Economic**					
GDP per capita (2005 PPP$)	0.35	0.70***	-0.42*	0.05	-0.47*
Gini index (2000,2005)	0.15	-0.20	0.02	0.29	-0.06
Foreign direct investment % of GDP (2000)	0.01	-0.08	-0.35**	-0.26	0.14
Employment in agriculture (%) (2005)	-0.16	-0.61***	0.41	0.06	0.48**
Labour force participation (2000)	-0.14	-0.51***	0.50*	-0.05	-0.06
**Social**					
HDI (2000 and 2005)	0.53***	0.55***	-0.64**	0.29*	-0.56**
Mobile telephone subscribers 2000	0.50***	0.66***	-0.44**	0.18	-0.38
Literacy (2000)	0.42***	0.42***	-0.74***	0.25*	-0.67***
Mean years of schooling (2005)	0.44**	0.50***	-0.24	0.27	-0.28
Homicide Rate (2005)	-0.42***	-0.03	0.15	-0.26*	-0.04
Political Rights (2010)	0.60***	0.15	-0.26	0.44***	0.05
Civil Liberties (2010)	0.64***	0.26	-0.39**	0.38***	-0.14
**Demographic**					
Population growth rate (2000–2005)	0.18	-0.06	0.03	0.34	0.29
Life expectancy at birth (2000–2005)	0.39*	0.54***	-0.62**	0.17	-0.51***
Total Fertility Rate (200–2005)	-0.18	-0.52***	0.42*	0.10	0.45**
Median age (2000)	0.31	0.35	-0.33*	0.01	-0.59***
Mean age at first union (2000)	0.29	0.45**	-0.55**	-0.01	-029
International net migration rate (2000–2005)	0.47***	0.35	-0.26	0.40***	-0.28
Remittances as percent of % GDP (2000)	-0.55***	-0.42**	0.68***	-0.36**	-0.13

Significance levels were derived from bootstrapped confidence intervals using 1000 replicates.

* correlation significant at the 10% level,

** 5% level and

*** 1% level

### Cluster 1: High intensities, late ages at migration, and low friction of distance

Membership of Cluster 1 is positively associated with political rights, civil liberties and net international migration (Table 4), all of which also return positive and statistically significant associations with overall migration intensities (Table 5), posting correlation coefficients ranging from 0.47 to 0.64. This suggests that the institutional framework, through high levels of social and political openness, may facilitate population movement. The three countries in this cluster also stand out as the only ones in our sample, with the exception of Venezuela, that receive net population gains, albeit small, from international migration. The positive association between migration intensities and net international migration (Table 5) suggests the two forms of movement are functionally connected, whereby international population gains either trigger or replace internal flows, further contributing to high migration intensities in Chile, Costa Rica and Panama. Membership of Cluster 1 is also positively associated with mean years of schooling (0.45), which returns a positive association with age at the migration peak (0.50). This suggests that the late migration peak in Chile, Costa Rica and Panama reflects a postponement in the completion of education to older ages compared with other Latin American countries. Evidence from countries around the world reveals that migration age patterns are shaped by the timing of four key life-course transitions: completing education, entering the labour market, forming a union and a birth of a child [55]. In the case of Cluster 1, however, there is no significant association with mean ages at first union, which suggests that migration in Chile, Costa Rica and Panama may be driven by education and employment-related motives, rather than by family-related reasons.

### Cluster 2: Low intensities, early ages, unbalanced flows and high friction of distance

This cluster brings together countries at lower levels of development as indicated by a strong negative association of cluster membership with GDP per capita (-0.73), mobile phone subscriber rates (-0.72), literacy (-0.55) and HDI (-0.63). The latter two indicators return strong negative correlations with the β parameter in Table 5, suggesting that the higher friction of distance in these countries is the result of lower socio-economic development. Membership of Cluster 2 is also associated with low urbanization (-0.60), which returns a negative association with migration effectiveness (-0.50). With the exception of Peru, countries in Cluster 2 are at earlier stages of the urban transition than countries in other clusters [39], which is a likely explanation for unbalanced flows as measured by migration effectiveness. It is also consistent with the theoretical framework set out by Rees et al. [4] showing how migration shapes population redistribution during the process of national development. Membership of Cluster 2 is also positively associated with the share of total employment in agriculture (0.64). Seasonal migration is a common income diversification strategy in rural areas of developing countries [56], and countries with a very high share of total employment in agriculture, in particular Ecuador (31.4%), Honduras (36.9%) and Haiti (50.5%), report some of the lowest migration intensities. This suggests that seasonal circulation may act as a substitute for permanent migration among rural populations of Latin America, as farmers are tied to the land, driving migration intensities lower in the agriculture-oriented economies of Cluster 2. Countries in this cluster are also characterised by net losses through international migration but have a positive association with remittances. International labour migration is a major livelihood strategy in less developed countries, and these links suggest that international outflows may be substituting for, and thereby reducing, internal migration in Cluster 2. All countries in Cluster 2 feature young populations (average median age of 21 years), high total fertility rates (from 2.8 to 4.0), and mean ages at first union below 22 years, with the two latter returning a strong association with age at migration peak (Table 5). These results indicate that the young age profile of migrants reflects early transitions to adult roles in these countries.

### Cluster 3: Moderate intensities, intermediate ages, balanced flows, and moderate friction of distance

Cluster 3 registers the lowest average migration effectiveness (Table 3), indicating more spatially balanced flows and counter-flows and this is consistent with positive links to urbanization (0.39) and mobile phone penetration (0.50), both of which return a negative association with migration effectiveness (i.e. more balanced flows) in Table 5. This aligns with theoretical expectations of increasingly balanced flows and counter-flows as regional disparities decline with economic development and countries progress through the urban transition [4]. Because measures of regional inequality are not readily available, we reported instead measures of income inequality as measured by the Gini index, but it did not return an association with migration effectiveness (Table 5). Migration effectiveness is, however, associated with mobile phone penetration, which has been shown to contribute to reducing regional differences [57] by facilitating connections between regions. Cluster 3 countries such as Argentina and Uruguay combine some of the highest rates of mobile phone subscribers in Latin America and the lowest MEIs (more balanced flows), and stand in stark contrast to countries in Cluster 2, which display the highest MEIs and the lowest rates of mobile phone penetration. Countries in this cluster tend to be larger geographically, but areal size varies and does not return a statistically significant association with any dimension of migration. With regard to overall intensities and migration ages, countries in this cluster appear to occupy an intermediate position between Cluster 1 and Cluster 2. Argentina, however, combines low migration intensity and a late migration age profile that is similar to that of countries in Cluster 1. A possible explanation for its low migration intensity relative to countries with similar HDI, such as Chile, lies in its net losses through international migration, which may be substituting for, and thereby, reducing internal migration.

### Cluster 4: Low intensities, late ages, unbalanced flows, and moderate friction of distance

Cluster 4 is characterised by an unexpected combination of low migration intensities and late age profiles. Few national indicators are associated with membership of Cluster 4 because it encompasses diverse countries, with levels of development ranging from low in El Salvador to medium in Cuba and Venezuela, and high in Mexico. Different explanations for migration characteristics must therefore be sought for each country. Cuba and Venezuela stand out with the lowest levels of civil liberties and political rights among the 19 countries in our sample. The institutional and legal framework in which population movement takes place is known to constrain mobility choice and countries that restrict population movement tend to record lower migration intensities [58, 59]. Cuba is a case in point with the introduction in 1997 of a permit to move permanently to Havana province, which requires authorisation from the owner of the property in Havana and certification that the dwelling complies with building requirements [60]. Cuba’s restrictive regulatory framework is a likely explanation for its very low migration intensity relative to its level of development, underlining the way government interventions can disrupt migration-development interactions. Another mechanism appears to be at play in El Salvador and Mexico, where net population losses due to international migration are among the highest in the region, suggesting that international outflows may be substituting for, and thereby reducing, internal migration in these countries. In line with theoretical expectations, explanation for the late migration age profile of Cluster 4 must be sought in relation to the timing of life-course transitions [55]. Cuba records the lowest total fertility rate in the region and high average years of schooling on par with countries in Cluster 1, signalling late life-course transitions. Conversely, the late migration age profiles of Mexico, El Salvador and Venezuela do not appear to reflect the age structure of the life-course, since low educational attainment, high TFRs, and early ages at first union point to transitions to adult roles at young ages. This suggests that cultural norms or the broader socio-economic context alter the timing of migration to generate unique migration age profiles in these countries.

## Discussion and conclusions

This paper has taken a first step towards a more comprehensive view of population mobility by recognizing that migration is a multi-dimensional process that cannot be adequately summarised by single measures. It has sought to provide a broader conceptual framework, coupled with a more robust analytical approach, to understanding migration systems by bringing together four key dimensions of migration: intensity, age, impact and distance. We have rigorously quantified each dimension by using comparable, scale-free indicators and examined how a broad range of forces interact to shape different migration systems for 19 Latin American countries.

The paper has sought to enhance migration theory by building on Zelinsky’s mobility framework which sketched links between migration and development [6]. We have incorporated additional dimensions of migration not originally considered by Zelinsky, in particular age and distance, and show that these key dimensions vary systematically across countries to form distinct migration systems. We have recognized the diversity of drivers of migration by considering a wide range of explanatory factors encompassing geographic, demographic, economic and social conditions. In so doing, we have demonstrated that while migration is inextricably linked to development, the nexus is neither simple nor linear. Different facets of migration evolve in distinctive ways. Moreover, local contingencies intervene to create unique migration systems in particular groups of countries.

We have shown that each facet of population movement provides a somewhat different picture of migration in Latin America: the ranking of countries differs according to the dimension under scrutiny. Migration intensities vary from highs exceeding 35 per cent in Chile and Panama to lows of less than 10 per cent in Haiti and Venezuela. Migration peaks after 27 years of age in Argentina and Costa Rica but before age 21 in Honduras and Peru. The effectiveness of migration in redistributing population varies from 55 per cent in Haiti down to 15 per cent in Argentina and Costa Rica. The friction of distance is highest in Nicaragua and Bolivia and lowest in Cuba and Costa Rica. This comparison also highlighted characteristics that distinguish Latin America from the rest of the world, in particular the limited impact of migration in redistributing populations and the diversity of migration age profiles.

We identified four distinct migration clusters across Latin America that form a broad sequence of low intensities, young ages, unbalanced flows and high friction of distance in countries at lower levels of development and the inverse pattern in more developed countries with high intensities, late ages, balanced flows and low friction of distance. Evidence suggests some linearity with development since intensity, age, effectiveness and distance—all return a statistically significant association with HDI. The exception is migration impact which has a more complex relationship with development because one of its two components, migration effectiveness, first rises then falls as nations urbanise and regional disparities grow then diminish [4]. While all facets of population movement appear to evolve in tandem as development progresses, local contingencies sometimes distort the broad migration-development nexus and migration patterns cannot therefore be explained solely by reference to level of development as originally anticipated by Zelinsky [6]. For example, political control of movement in Cuba, and international migration losses from Argentina, El Salvador and Mexico constrain migration intensities relative to the level of national development. In countries with net international outflows or significant remittances from overseas, it appears that international outflows substitute for, and thereby reduce, internal migration. Thus, while there are clear archetypes at the ends of the development spectrum, migration patterns at intermediate levels of development are less clear-cut as local circumstances appear to distort the links between development and migration.

Despite these apparent anomalies, the analysis has highlighted specific sets of forces that shape the way particular aspects of migration are transformed during the process of national development and systematically differentiate countries in their mobility profile. Confining our attention to Latin America enabled us to explore variation in migration free of the large scale confounding effect of major differences in historical and cultural norms which hinder broader international comparisons. By minimizing these dissimilarities, we were able to show that it is the structure of the economy (level of agricultural employment and GDP per capita), technological progress (mobile phone penetration) and civil and political rights which largely underpin variations in migration intensity, the balance of flows and the friction of distance, whereas ages at union formation, fertility and levels of schooling give rise to systematic differences in the age profile of migration.

The conceptual and analytical approach adopted in this paper provides a general framework for examining migration systems and improving our understanding of the relationship between development and migration processes. It could also be applied to other regions of the world collecting comparable migration data and to single countries with sufficiently long time-series to explore migration trends. In some countries of North America and Europe, recent evidence points to declining migration intensities [61, 62], reduced migration impact [63] and delayed migration age patterns [50] suggesting the emergence of particular migration trajectories, but it remains unclear how these changes are being shaped by wider societal transformations. Economic, technological, political and social forces are doubtless at play, but may manifest differently from those identified in the particular context of Latin America. While the approach adopted here might usefully be applied to these alternative regional settings, non-permanent forms of mobility, such as seasonal migration and long distance commuting also invite consideration, since they may either complement, or substitute for, permanent forms of migration. Similar techniques are readily applicable, though suitable data are as yet wanting in most parts of the world.
